# Currency as a mobile environmental reservoir of bacterial pathogens in Africa: a systematic review and meta-analysis

**DOI:** 10.3389/fpubh.2026.1921421

**Published:** 2026-10-19

**Authors:** Chinyere Nkemjika Anyanwu, Paul-Chima Okechukwu Ugwu, Rajendra Neel, Swase Dominic Terkimbi, Michael Ben Okon, Zeinab Adam Abakar Mohamed, Onyinyechi Bede-Ojimadu, Emeka Godson Anyanwu

**Affiliations:** 1 Department of Microbiology and Immunology, Faculty of Biomedical Sciences, Kampala International University, Bushenyi, Uganda; 2 Department of Publication and Extension, Kampala International University, Bushenyi, Uganda; 3 Department of Pharmacy, Faculty of Health Sciences, Victoria University, Kampala, Uganda; 4 Department of Medical Laboratory Science, School of Allied Health Sciences, Kampala International University, Bushenyi, Uganda; 5 Department of Anatomy, Faculty of Biomedical Sciences, Kampala International University, Bushenyi, Uganda

**Keywords:** Africa, bacterial contamination, banknotes, currency contamination, environmental reservoirs, public health

## Abstract

**Background:**

Circulating currency is among the most frequently handled objects in daily life and may facilitate microbial transfer between individuals, food products and environmental surfaces. Although bacterial contamination of currency has been documented across Africa, the overall burden and epidemiological significance of this contamination have not been systematically quantified. This systematic review and meta-analysis synthesized available evidence on bacterial contamination of circulating currency in Africa.

**Methods:**

PubMed, Scopus, Web of Science, African Journals Online (AJOL), Google Scholar were systematically searched. Studies investigating bacterial contamination of circulating currency notes in African countries were eligible. Random-effects meta-analysis was performed to estimate pooled contamination prevalence and organism-specific prevalence while narrative synthesis was used for outcomes unsuitable for quantitative pooling.

**Results:**

Forty-six studies met the inclusion criteria and contributed to the systematic and narrative synthesis. Of these, 12 studies involving 2,003 currency notes provided sufficient comparable data for the meta-analysis of overall bacterial contamination prevalence. The pooled bacterial contamination prevalence was 79.3% (95% CI: 60.9–90.4%). Organism-specific analyses showed that *Staphylococcus aureus* accounted for 31.2% (95% CI: 24.7–38.4%) and *Escherichia coli* for 24.0% (95% CI: 17.8–31.5%) of bacterial isolates recovered from contaminated currency. Narrative evidence additionally identified *Klebsiella*, *Salmonella*, *Pseudomonas*, *Proteus*, and *Shigella* species among frequently reported contaminants. Several studies also documented multidrug-resistant, extensively drug-resistant, and pan drug-resistant bacterial strains. Lower-denomination and physically worn banknotes exhibited higher contamination burdens than higher-denomination or newer notes.

**Conclusion:**

Bacterial contamination of circulating currency is highly prevalent across African settings and includes clinically important and antimicrobial-resistant bacterial species. These findings suggest that circulating currency may serve as an underrecognized environmental reservoir and a potential interface for microbial exchange between human, food, and environmental systems. From a One Health perspective, strengthening hand hygiene, discouraging simultaneous handling of food and money and improving currency management may help reduce contamination risks and limit microbial dissemination within communities.

## 1 Introduction

Currency notes are among the most commonly handled objects in daily life repeatedly moving between individuals, occupations and environments. Their widespread circulation and repeated contact with hands, food products and contaminated surfaces make them potential fomites capable of acquiring and disseminating microorganisms (1, 2). Unlike fixed environmental surfaces, circulating currency repeatedly traverses households, markets, transportation systems, healthcare facilities, food vending environments and commercial settings. This creates opportunities for microbial transfer across diverse human and environmental interfaces (3–5). Consequently, currency has increasingly attracted attention as a potential environmental reservoir of microorganisms with implications for public health and hygiene.

The subject may be particularly important in African settings where cash-based transactions remain common within informal economies and where food handling, transportation and commercial activities often occur under conditions that may facilitate microbial transfer (6). Additional factors such as prolonged circulation of worn banknotes, simultaneous handling of food and money, overcrowded market environments and limited sanitation infrastructure may further increase opportunities for microbial contamination and persistence on currency surfaces (5, 7, 8). As a result, circulating currency may serve as a frequently overlooked vehicle for microbial exchange within community and occupational settings.

Studies conducted across different African regions have reported the recovery of clinically relevant organisms, including *Staphylococcus aureus*, *Escherichia coli*, *Klebsiella* spp., *Salmonella* spp., *Pseudomonas* spp., and *Shigella* spp., from circulating currency notes (7, 9–14). The presence of both skin-associated and enteric bacteria suggests multiple contamination pathways involving direct human contact, food handling, and fecal contamination. Given the substantial burden of infectious diseases, foodborne illnesses, healthcare-associated infections and antimicrobial resistance across many African countries (15, 16), contaminated currency may represent an underappreciated environmental interface linking community, food and healthcare-associated microbial exposures. From a One Health perspective, circulating currency may function as a point of interaction between human, food, and environmental microbial reservoirs, facilitating microbial exchange across otherwise distinct ecological niches.

Despite growing interest in microbial contamination of currency, the available African evidence remains geographically fragmented and methodologically heterogeneous. Existing studies vary considerably in sampling strategies, microbiological identification methods, laboratory procedures and reporting practices, making direct comparisons challenging. Although numerous investigations have documented bacterial contamination of currency in individual countries and local settings, no previous systematic review and meta-analysis has quantitatively synthesized continent wide evidence to estimate pooled contamination prevalence, characterize dominant bacterial contaminants or evaluate organism-specific prevalence patterns across African currencies. Consequently, the overall burden, distribution and public health significance of bacterial contamination of circulating currency in Africa remain incompletely understood.

Therefore, this systematic review and meta-analysis was undertaken to synthesize available evidence on bacterial contamination of circulating currency in Africa. Specifically, the review aimed to estimate the pooled prevalence of bacterial contamination, identify the predominant bacterial species recovered from contaminated currency and characterize contamination patterns with potential public health relevance. By integrating quantitative and narrative evidence, this review provides a comprehensive assessment of the epidemiology of currency-associated bacterial contamination and its implications for hygiene, food safety, and public health in African settings.

## 2 Methods

### 2.1 Study design and reporting framework

This study was conducted as a systematic review and meta-analysis to synthesize evidence on the prevalence of bacterial contamination of circulating currency notes in African settings. The review methodology and reporting strategy followed in this review were based on the Preferred Reporting Items for Systematic Reviews and Meta-Analyses (PRISMA 2020) guidelines and recommendations on transparent reporting of quantitative evidence synthesis (17). Both narrative and quantitative synthesis approaches were used to comprehensively assess the prevalence of contamination and organism-specific distribution patterns in contaminated banknotes across Africa.

### 2.2 Eligibility criteria

Studies were considered eligible if they investigated bacterial contamination of circulating paper or polymer currency notes within African countries and reported microbiological isolation and identification of bacteria. Observational study designs including cross-sectional studies, environmental microbiology investigations, and laboratory-based surveillance studies were included provided they contained extractable quantitative microbiological data such as sample sizes, contamination frequencies or organism-specific isolate counts. Only studies published in English were included because resources for reliable translation and independent verification of non-English full texts were not consistently available during the review. There was no year restriction. Studies were excluded if they were conducted outside Africa or investigated non-African currencies. We also excluded studies assessing non-circulating, experimentally inoculated currency as well as studies focused exclusively on non-bacterial contaminants such as fungi, viruses, or parasites without reporting bacterial outcomes. Reviews, editorials, commentaries, protocols, conference abstracts without sufficient extractable data and case reports were also excluded. Although some studies were not eligible for the quantitative synthesis, they were included in the review for context and narrative reasons especially in the background, introduction, and discussion sections of the review. These studies contributed additional epidemiological, methodological and public health information pertinent to the study of bacterial contamination of circulating currencies in Africa.

### 2.3 Information sources and search strategy

A comprehensive literature search was conducted in PubMed, Scopus, Web of Science, African Journals Online (AJOL) and Google Scholar. The final electronic search was conducted on 20th April 2026. The search strategy was developed around the core concepts using a combination of Boolean operators: (i) circulating currency and (ii) bacterial or microbial contamination. To enhance retrieval of geographically indexed studies, both continent-level descriptors (Africa, African) and individual African country names were incorporated into the search strategy, as many eligible studies reported country-specific findings without explicitly indexing the broader term “Africa” in titles, abstracts or keywords. Search syntax was adapted to the indexing structure and functionality of each database. In addition, the reference lists of eligible articles and relevant reviews were manually screened to identify further studies. The complete database specific search strategies are provided in Supplementary Table S1.

### 2.4 Screening and selection

The selected articles were de-duplicated before screening. Titles, abstracts and full-text articles were independently screened by two reviewers using predefined eligibility criteria. Discrepancies in study selection, eligibility assessment or data extraction were resolved through discussion and re-evaluation of the original article. Where disagreements persisted, a third reviewer was consulted to achieve consensus. This process was implemented to minimize selection bias and ensure consistency throughout the review. The study selection process is summarized in the PRISMA flow diagram (Figure 1) showing records identified, screened and included.

**Figure 1:**
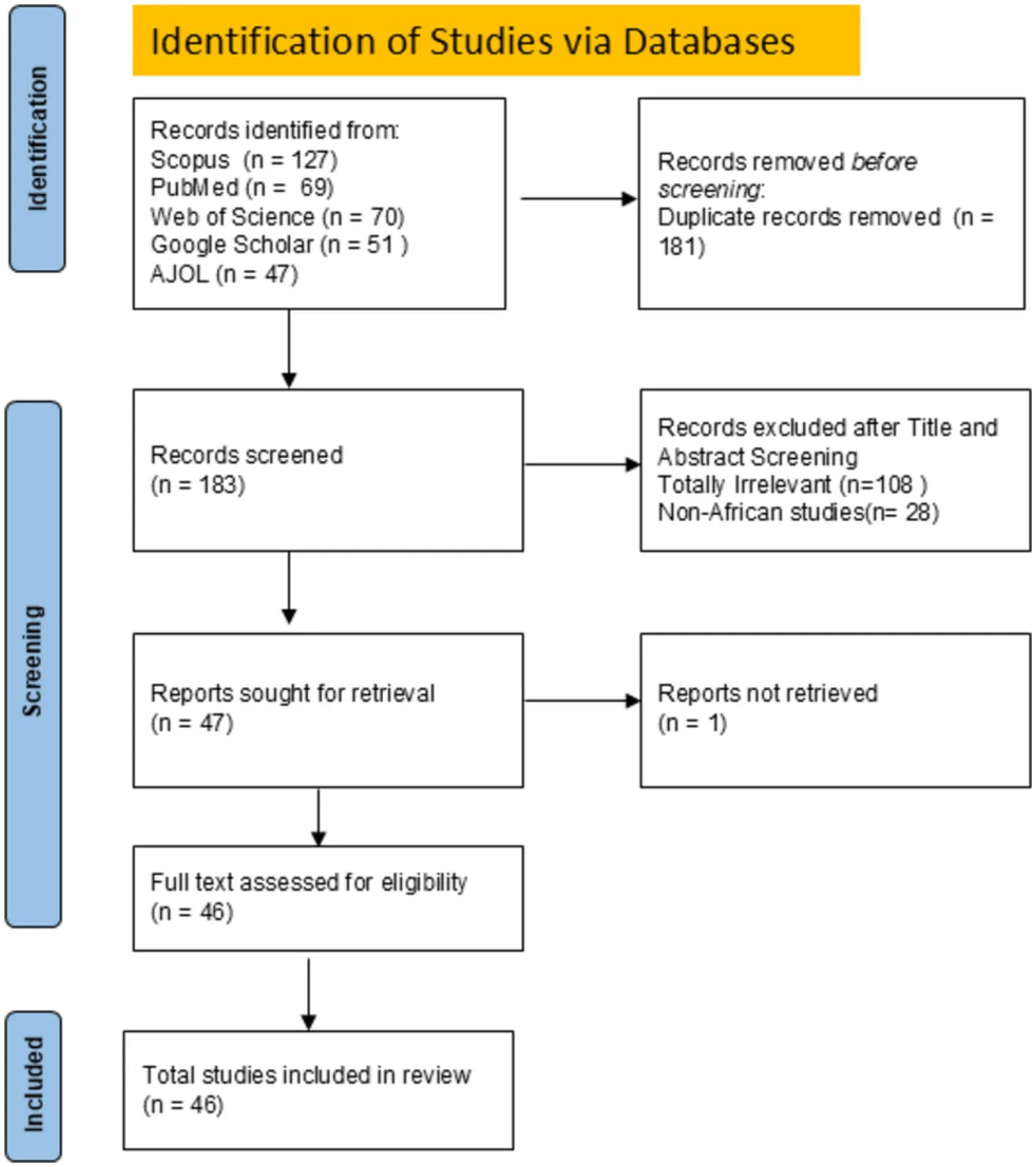
PRISMA flow diagram for systematic review.

### 2.5 Data extraction

A standard data extraction template was developed and used to extract the data. Data extracted included author, year of publication, country. Study design, sample size, number of contaminated notes, bacterial species isolated, organism-specific isolate prevalence.

### 2.6 Quality assessment

The methodological quality and risk of bias of included studies were assessed using the Joanna Briggs Institute (JBI) Critical Appraisal Checklist for prevalence studies (18). Each domain of the JBI checklist was rated as “Yes,” “No,” “Unclear,” or “Not Applicable” where appropriate. For overall study quality assessment, studies with 7–9 domains rated as “Yes” were classified as having a low risk of bias, those with 4–6 domains rated as “Yes” were considered to have a moderate risk of bias and studies with 0–3 domains rated as “Yes” were classified as having a high risk of bias. Assessments of risk of bias were summarized using traffic light plots generated with the robvis visualization tool as shown in Supplementary Figure S1.

Publication bias was assessed using funnel plots and Egger’s regression test implemented in the Metafor package in R software (version 2024.12.1). A *p*-value < 0.05 was considered suggestive of statistically significant small-study effects and possible publication bias.

### 2.7 Quantitative synthesis and statistical analysis

Quantitative synthesis was also performed using R statistical software within the RStudio environment using the metafor package. Random-effects meta-analysis models were applied to account for anticipated methodological and epidemiological heterogeneity across the included studies. Pooled prevalence estimates and corresponding 95% confidence intervals (95% CI) were calculated using inverse variance weighting following logit transformation of proportions to stabilize variance across studies. Statistical heterogeneity was evaluated using Cochran’s *Q* statistic, between-study variance (*τ*^2^), and Higgins’ inconsistency statistic (*I*^2^), with *I*^2^ values of 25%, 50%, and 75% interpreted as low, moderate, and high heterogeneity, respectively. Organism-specific subgroup meta-analyses were conducted where sufficient extractable data were available. Forest plots were generated to visualize pooled prevalence estimates, confidence intervals, study weights and between-study variability. Leave-one-out analysis was undertaken to examine the effect of the omission of each individual study on prevalence estimates, between-study heterogeneity and pooled prevalence estimates.

### 2.8 Narrative synthesis

Studies that could not be quantitatively synthesized were analyzed using narrative synthesis. This approach was applied to studies with substantial methodological heterogeneity, variations in reporting formats, insufficient quantitative data, or a lack of extractable numerators and denominators required for meta-analysis.

## 3 Results

### 3.1 Characteristics of included studies

A total of 46 studies met the eligibility criteria and were included in the review. Studies were conducted across 10 African countries with Nigeria contributing the largest proportion of evidence. The included studies demonstrated substantial geographic and methodological diversity across the African continent. Eligible studies were conducted between 2007 and 2026 and originated predominantly from Nigeria, Ethiopia, Ghana, Sudan, Egypt, South Africa, the Democratic Republic of Congo, South Sudan, Kenya and Tanzania, representing West, East, North, Central, and Southern Africa. The geographical distribution of the evidence base is reflected by studies conducted in diverse settings across these countries (12–14, 19–25). Although studies were identified from all five African regions, the evidence base was geographically uneven, with a substantial concentration of studies in West and East Africa.

Sample sizes varied considerably across studies ranging from fewer than 20 currency notes in small exploratory investigations to 624 notes in large-scale studies. Currency samples were collected from a wide range of circulation settings including open markets, food vending environments, butcher shops, restaurants, public transportation systems, hospitals, banks, university campuses and other community commercial centers. Several studies specifically focused on high-contact occupational settings such as food vendors, fish sellers, meat handlers, market traders, banking environments, healthcare facilities, universities and transportation networks (26–36).

Microbiological analyses across the included studies predominantly relied on conventional culture-based bacteriological methods using selective and differential media, often complemented by Gram staining, biochemical identification and antimicrobial susceptibility testing. Frequently identified bacterial genera included *Staphylococcus, Escherichia, Klebsiella, Salmonella, Pseudomonas, Proteus, Shigella, Bacillus*, and *Enterobacter* species. A smaller number of studies incorporated molecular techniques, including polymerase chain reaction (PCR), 16S rRNA gene sequencing and phylogenetic analyses, while others focused on specific bacterial groups, resistant phenotypes, parasitic contaminants or denomination-related contamination patterns (37–44). Detailed study characteristics are presented in Supplementary Table S2 while a summary of the evidence base is provided in Table 1.

**Table 1:** Summary of study characteristics.

Domain	Category	No. of studies (%)
Geographic distribution	West Africa	31 (67.4)
	East Africa	8 (17.4)
	North Africa	5 (10.9)
	Southern Africa	1 (2.2)
	Central Africa	1 (2.2)
Countries represented	Nigeria	28 (60.9)
	Ethiopia	5 (10.9)
	Sudan	4 (8.7)
	Ghana	3 (6.5)
	Kenya	1 (2.2)
	Egypt	1 (2.2)
	South Africa	1 (2.2)
	Tanzania	1 (2.2)
	South Sudan	1 (2.2)
	Democratic Republic of Congo	1 (2.2)
Currency type investigated	Paper banknotes only	42 (91.3)
	Coins only	2 (4.3)
	Notes and coins	2 (4.3)
Sample size distribution	<100 samples	20 (43.5)
	100–199 samples	15 (32.6)
	200–399 samples	5 (10.9)
	≥400 samples	4 (8.7)
	Not Reported	2(4.3)
Predominant study settings	Markets and traders	22 (47.8)
	Food vendors, restaurants and butcheries	18 (39.1)
	Hospitals and healthcare facilities	8 (17.4)
	Transportation systems	7 (15.2)
	Banks	4 (8.7)
	Universities and community settings	5 (10.9)
Laboratory approaches	Culture and biochemical identification	46 (100)
	Antimicrobial susceptibility testing included	15 (32.6)
	Molecular characterization (PCR/16S sequencing)	4 (8.7)
Most frequently reported organisms	*Staphylococcus aureus*	30 studies
	*Escherichia coli*	29 studies
	*Klebsiella* spp.	18 studies
	*Salmonella* spp.	16 studies
	*Pseudomonas* spp.	15 studies
	*Proteus* spp.	8 studies
	*Shigella* spp.	7 studies
	*Enterobacter* spp.	5 studies

PCR, Polymerase Chain Reaction; 16S rRNA, 16S ribosomal ribonucleic acid. Categories are not mutually exclusive; individual studies may contribute to more than one setting, laboratory approach, or organism category.

### 3.2 Risk of bias assessment

The risk-of-bias assessment shows a generally good methodological quality among the included studies. Most studies were classified as having a low overall risk of bias with low-risk ratings observed across the majority of JBI domains. Methodological concerns were primarily related to sampling procedures and sample size adequacy which were rated as high or unclear risk in a limited number of studies. The evidence base demonstrated satisfactory methodological rigor, supporting the reliability of the findings synthesized in this review. A detailed domain level risk-of-bias assessment for all included studies is presented in Supplementary Figure S1.

### 3.3 Overall prevalence of bacterial contamination on currency notes

Although 46 studies met the eligibility criteria for the systematic review, only studies reporting sufficient quantitative data were eligible for meta-analysis. Consequently, 12 studies were included in the pooled prevalence analysis.

Across the 12 studies included in the meta-analysis, 1,455 of 2,003 currency notes were contaminated, corresponding to a crude aggregate proportion of 72.6%. When study-specific prevalence estimates were synthesized using a random-effects model, the pooled prevalence of bacterial contamination was 79.3% (95% CI: 60.9–90.4%; Figure 2).

**Figure 2:**
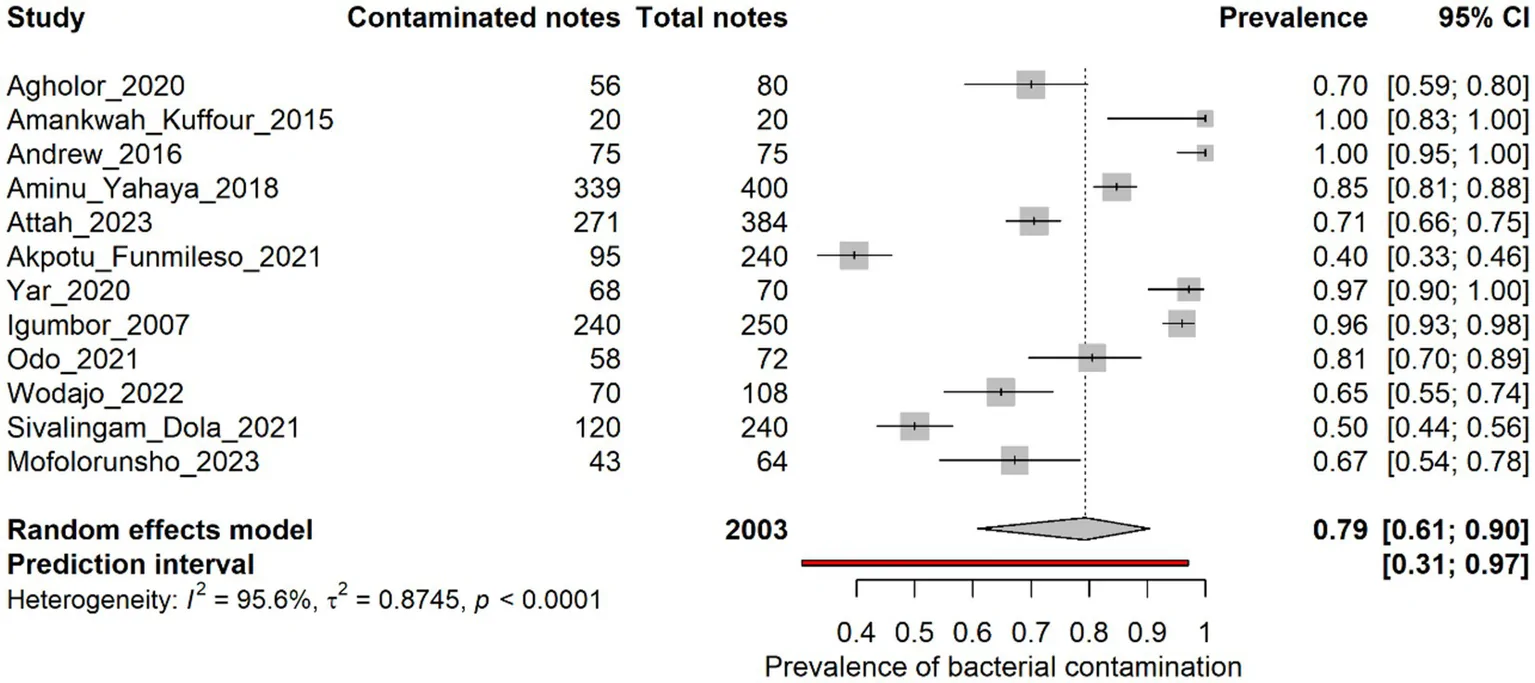
Forest plot of pooled bacterial contamination prevalence.

Individual study prevalence estimates ranged from 39.6% in Nigeria by Akpotu and Funmileso (45), to 100% in studies conducted in Ghana and South Sudan by Amankwah-Kuffour et al. and Andrew, respectively (9, 46). Although studies reporting complete contamination generally involved relatively small sample sizes. Substantial between-study heterogeneity was observed (*I*^2^ = 95.6%, *Q* = 252.06, *p* < 0.0001), indicating considerable variability in prevalence estimates across studies. Such heterogeneity is expected in environmental microbiology investigations conducted across diverse geographical locations, circulation settings, sampling strategies, and laboratory methodologies.

#### 3.3.1 Publication bias assessment

Visual inspection of the funnel plot suggested mild asymmetry among the included studies (Figure 3). However, Egger’s linear regression test did not demonstrate statistically significant evidence of small-study effects or publication bias (*t* = 1.78, *p* = 0.1053).

**Figure 3:**
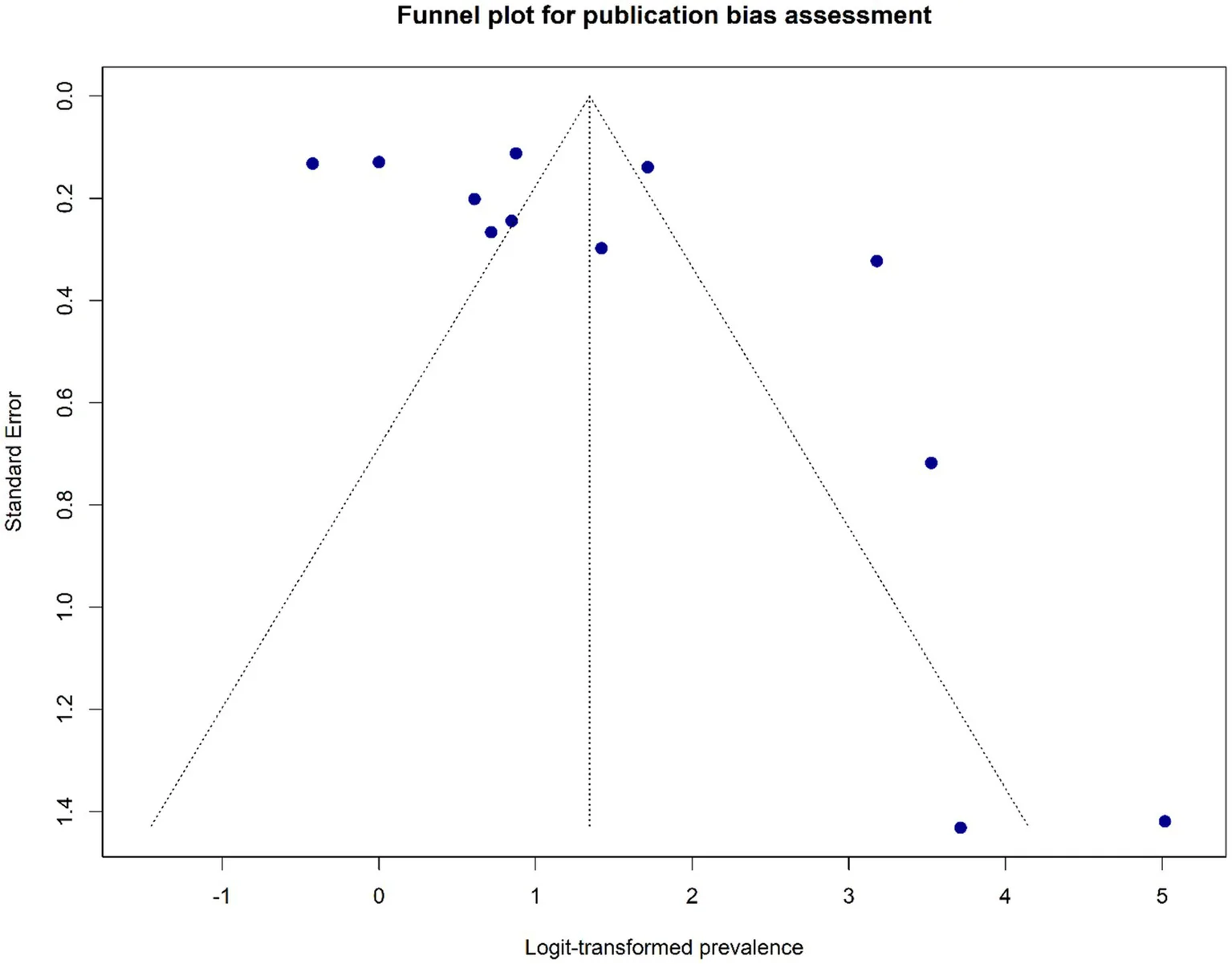
Funnel plot for overall contamination prevalence.

#### 3.3.2 Sensitivity analysis

Leave-one-out sensitivity analysis demonstrated that the pooled contamination prevalence estimate remained relatively stable following sequential omission of individual studies. The pooled prevalence ranged from 75.2% (95% CI: 55.9–87.9%) after exclusion of Igumbor et al. (12) to 81.9% (95% CI: 63.4–92.2%) following omission of Sivalingam and Dola (47). The leave-one-out sensitivity analysis is as shown in Figure 4.

**Figure 4:**
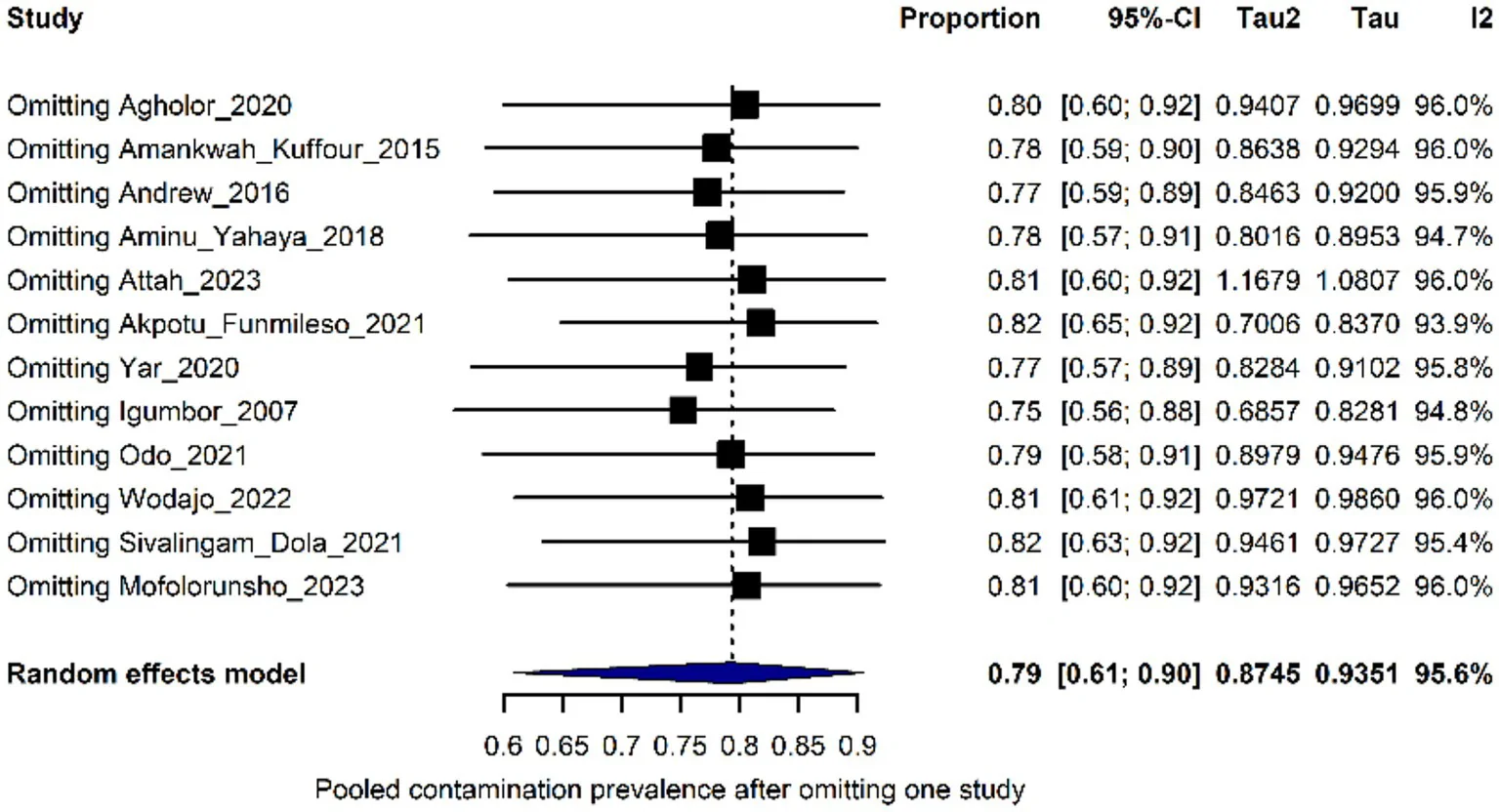
Leave-one-out sensitivity analysis for overall contamination prevalence.

Substantial heterogeneity persisted throughout the sensitivity analyses (*I*^2^ range: 93.9–96.0%). Nevertheless, the pooled prevalence estimate remained remarkably stable following sequential omission of individual studies, varying only from 75.2 to 81.9%. This finding indicates that no single study exerted a disproportionate influence on the overall estimate, thereby supporting the robustness of the meta-analytic findings.

### 3.4 Organism-specific prevalence of bacteria isolated from currency notes

Organism-specific meta-analyses were performed only for *Staphylococcus aureus* and *Escherichia coli*, as these were the only bacterial species reported with sufficient frequency and comparable quantitative data across studies to permit reliable pooled estimation. The total number of bacterial isolates included in each organism-specific meta-analysis varied according to the availability of species-specific isolate data reported by the included studies. Consequently, denominator values differed slightly across analyses.

#### 3.4.1 Staphylococcus aureus

For organism-specific analyses, the denominator represented the total bacterial isolates reported in the contributing studies rather than the number of currency notes examined. Accordingly, the 1,811 isolates in the *S. aureus* analysis represent the aggregate bacterial-isolate denominator reported across the 10 studies with extractable and comparable *S. aureus* data.

A random-effects meta-analysis of 10 studies comprising 1,811 bacterial isolates demonstrated that *Staphylococcus aureus* accounted for a pooled prevalence of 31.2% (95% CI: 24.7–38.4%) among bacterial isolates recovered from contaminated currency notes (Figure 5). Individual study estimates ranged from 17.7 to 48.6%, indicating substantial variation across study settings. Considerable heterogeneity was observed (*I*^2^ = 77.5%, *Q* = 39.92, *p* < 0.0001). Egger’s regression test showed no evidence of small-study effects or publication bias (*t* = −1.06, *p* = 0.3213). Sensitivity analysis demonstrated stable pooled estimates ranging from 29.8 to 32.9%, (Figure 6), indicating that no individual study disproportionately influenced the overall findings.

**Figure 5:**
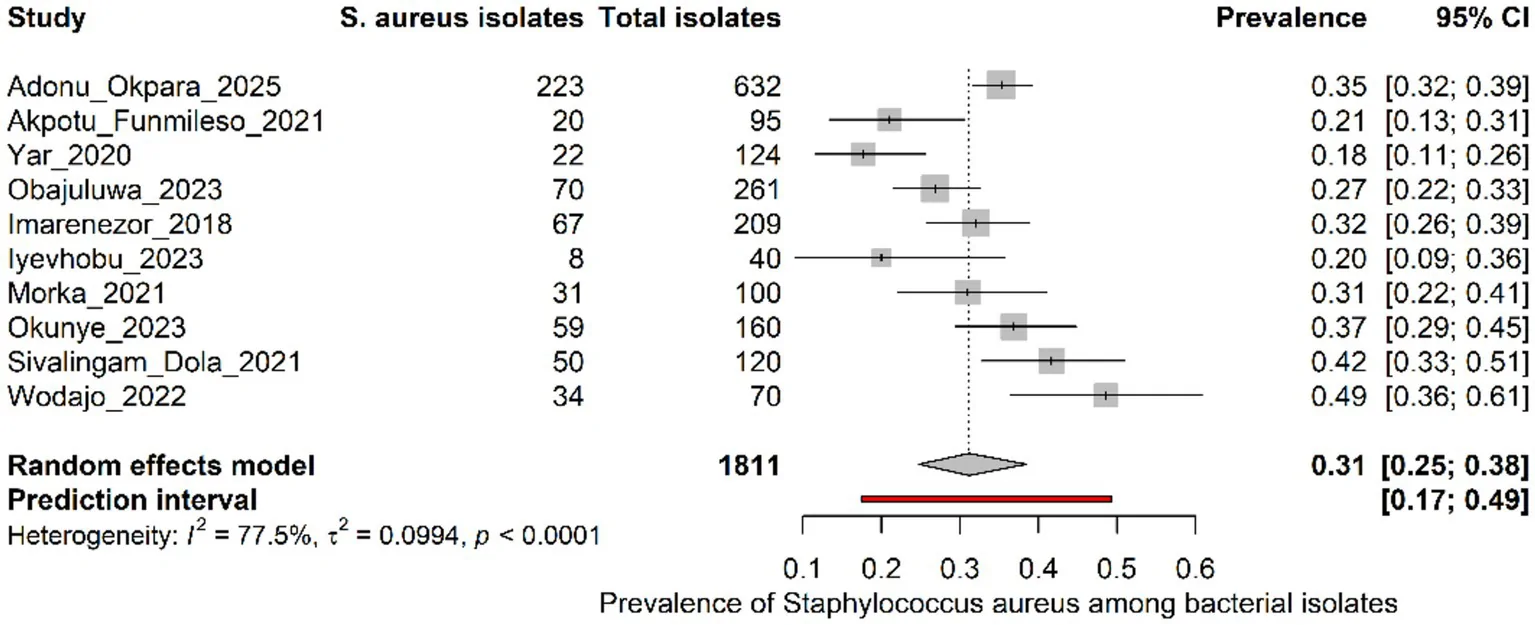
Forest plot of pooled *S. aureus* prevalence.

**Figure 6:**
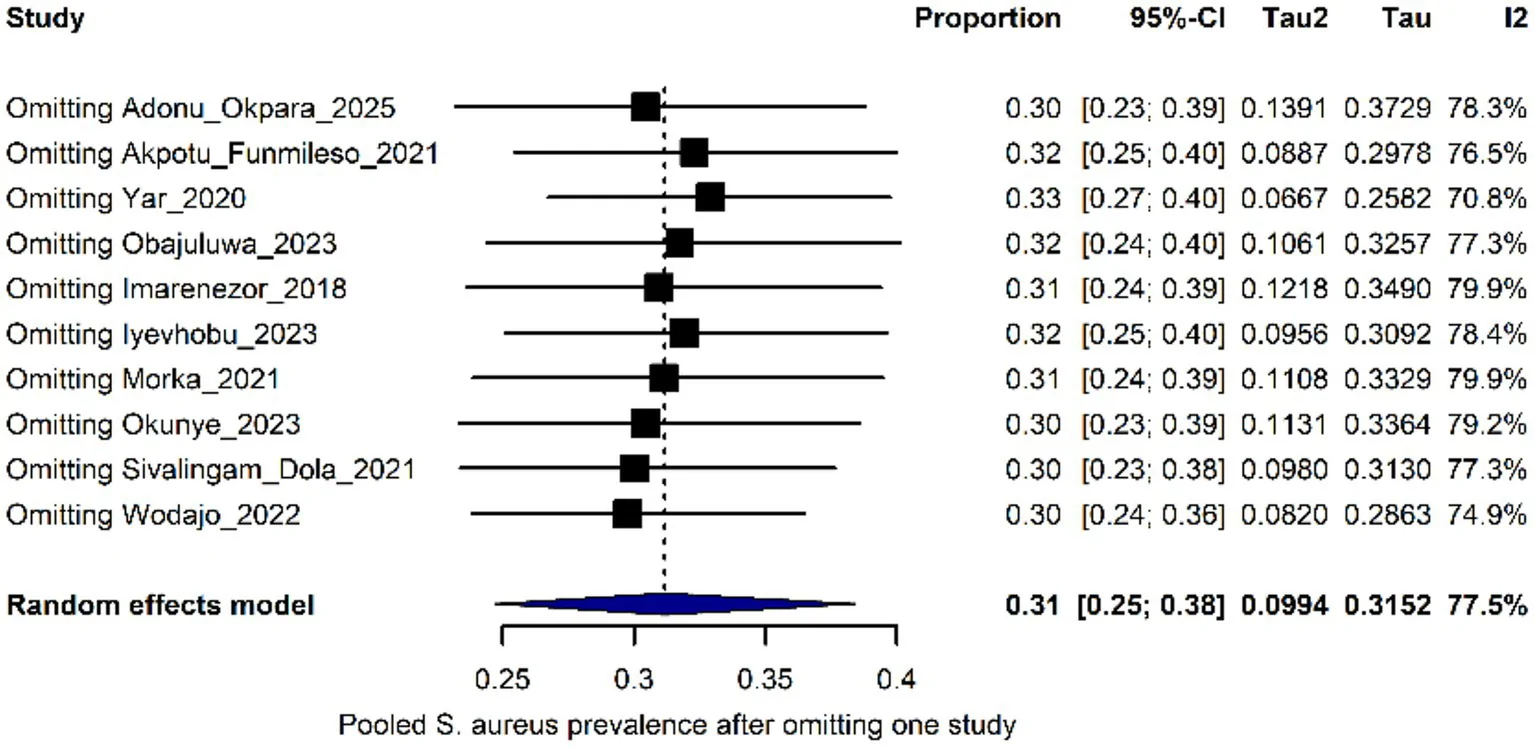
Leave-one-out sensitivity analysis for *S. aureus* prevalence.

#### 3.4.2 Escherichia coli

A random-effects meta-analysis involving 10 studies and 1,784 bacterial isolates demonstrated that *Escherichia coli* accounted for a pooled prevalence of 24.0% (95% CI: 17.8–31.5%) among bacterial isolates recovered from contaminated currency notes (Figure 7). Individual study estimates ranged from 11.0 to 44.2%, reflecting substantial variability across studies. Considerable heterogeneity was identified (*I*^2^ = 86.3%, *Q* = 65.90, *p* < 0.0001). Egger’s regression test showed no statistically significant evidence of publication bias (*t* = 1.84, *p* = 0.1037). Sensitivity analysis yielded pooled prevalence estimates ranging from 22.4 to 25.9%, supporting the robustness of the pooled estimate as shown in Figure 8.

**Figure 7:**
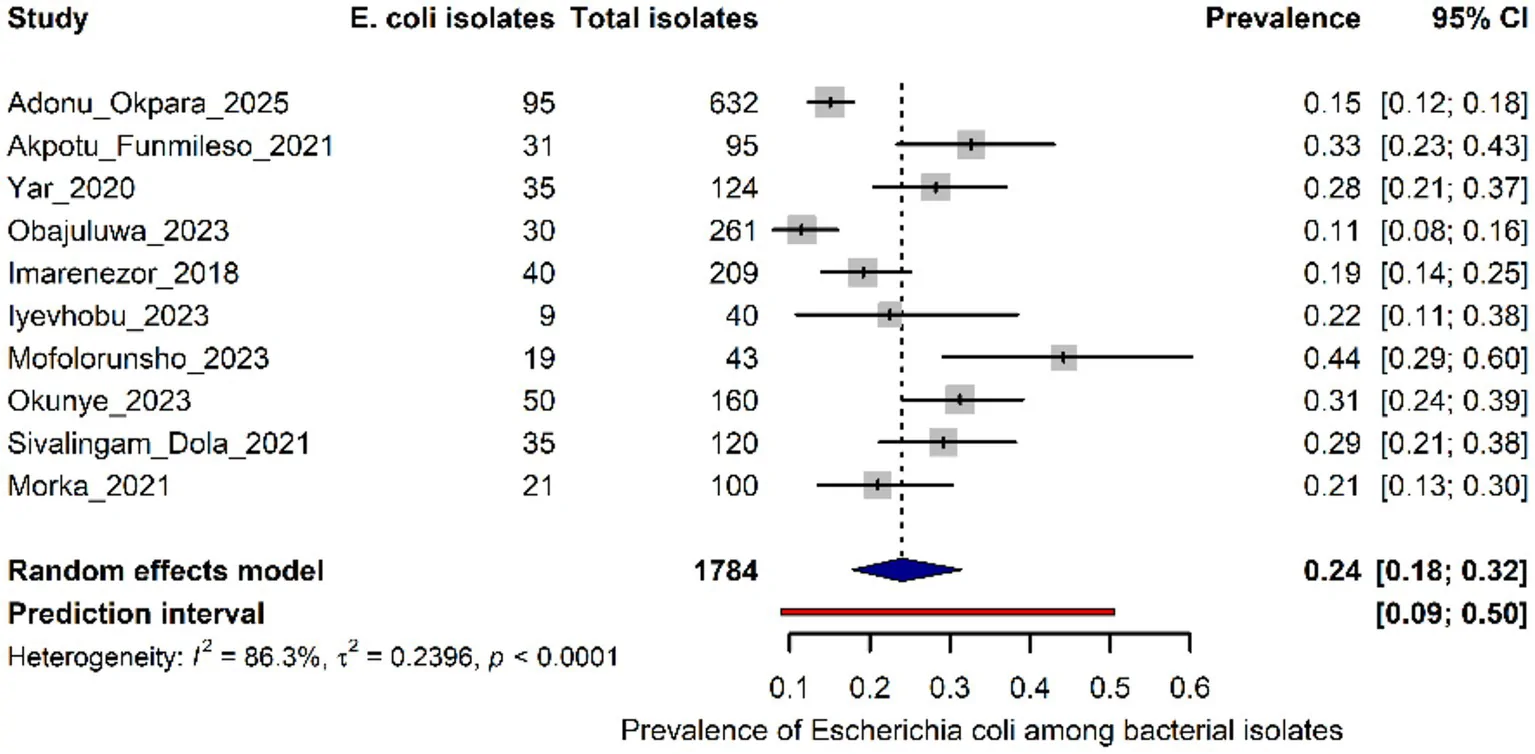
Forest plot of pooled *E. coli* prevalence.

**Figure 8:**
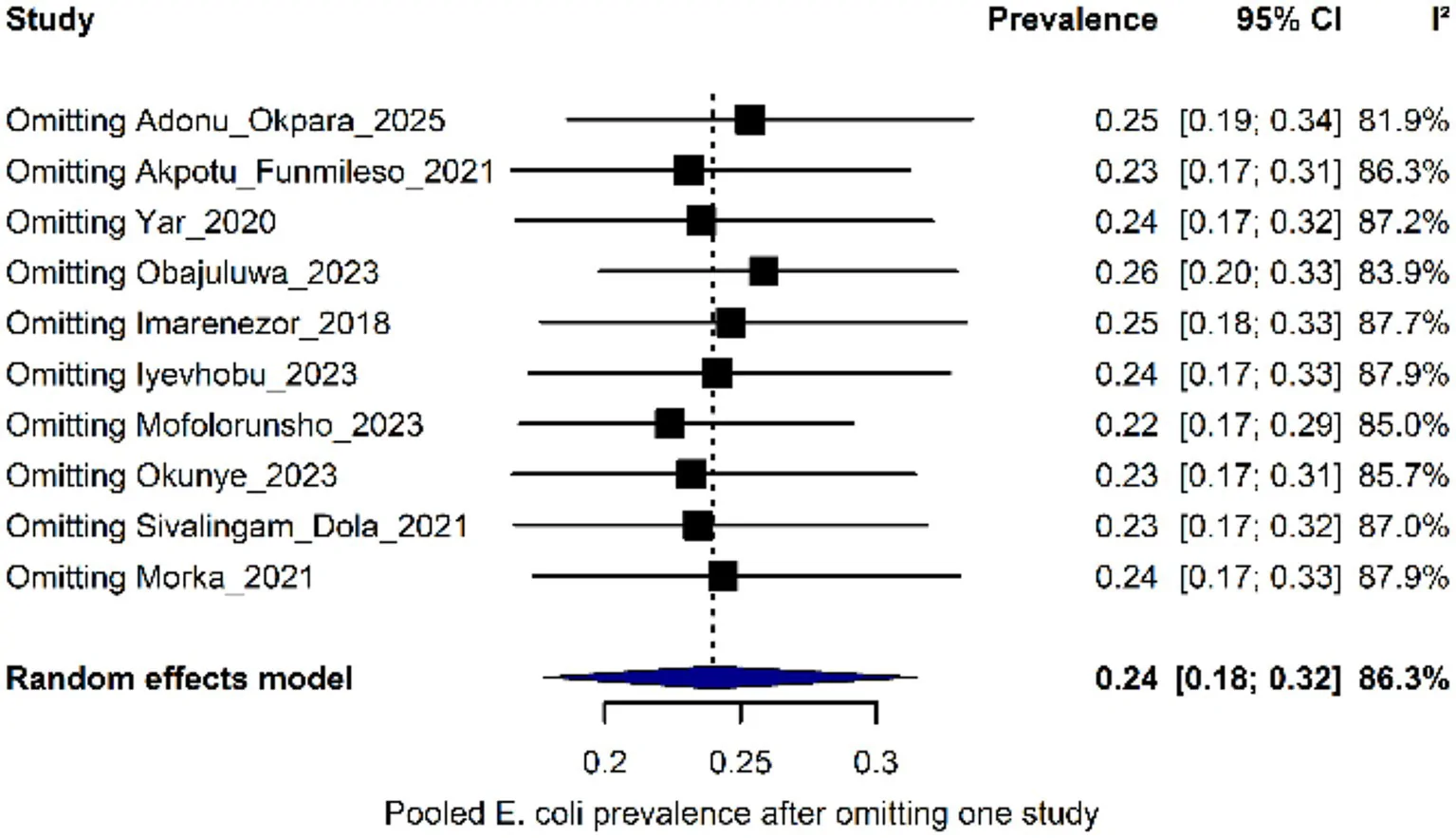
Leave-one-out sensitivity analysis for *E. coli* prevalence.

### 3.5 Antimicrobial resistance profiles of bacteria isolated from currency notes

While the primary focus of this study was bacterial contamination prevalence, incidental findings relating to antimicrobial resistance patterns were identified in several studies and are presented because of their possible implications for community level transmission of resistant organisms. Antimicrobial susceptibility results showed a high prevalence of resistance among recovered bacterial isolates, including multidrug-resistant (MDR; resistance to ≥3 antimicrobial classes), extensively drug-resistant (XDR; susceptibility retained to only one or two classes) and pan drug-resistant (PDR; resistance to all tested antimicrobial classes) strains (7, 47–51).

Ampicillin, penicillin, tetracycline, cotrimoxazole, erythromycin and related β-lactam antibiotics were the most consistent resistance observed (7, 47, 51). However, a few studies reported comparatively lower resistance rates and retained susceptibility to ciprofloxacin, gentamicin and other aminoglycosides among selected bacterial isolates, suggesting that these agents may still retain partial therapeutic activity against some currency-associated bacterial populations despite the broader burden of multidrug resistance. (47, 52). In Nigeria, Ghana, Ethiopia, Sudan, and Egypt, clinically important resistant phenotypes including methicillin-resistant *Staphylococcus aureus* (MRSA), multidrug-resistant enteric bacteria, resistant *Klebsiella* spp. and multidrug-resistant *Pseudomonas aeruginosa* have been reported from currency-associated and related environmental isolates, highlighting the potential role of contaminated banknotes in environmental dissemination of antimicrobial resistance (11, 50, 52, 53).

### 3.6 Denomination, material and occupation related contamination patterns

In various studies, it was found that lower denomination notes had higher contamination load than higher denomination notes (7, 9, 11, 46). Older and physically worn-out currency notes were consistently associated with higher levels of bacterial contamination compared with newer or cleaner notes (7, 9, 21, 41, 46).

Some studies additionally compared contamination patterns between polymer-based and traditional paper currency notes and generally reported lower microbial contamination burdens on polymer notes (30, 54). Occupational differences in contamination burden were also observed with currency notes obtained from food vendors, butchers, slaughterhouses and meat handlers demonstrating substantially higher bacterial contamination compared with notes collected from transport operators, market traders and banking environments (9, 10).

## 4 Discussion

This systematic review and meta-analysis show that contaminated currency notes represent a plausible but underrecognized environmental interface within the various African communities. Rather than functioning as isolated contaminated objects, banknotes may act as mobile contact surfaces that repeatedly connect hands, foods, commercial spaces and public environments.

The pooled contamination prevalence of 79.3% suggests that bacterial contamination of circulating currency is widespread across African settings. Although contamination prevalence varied considerably between studies, the overall estimate indicates that exposure to contaminated currency surfaces is likely a routine occurrence in many community and occupational environments. This prevalence is consistent with the notion that banknotes function as frequently exchanged environmental surfaces capable of accumulating microbial contaminants during routine circulation. Similarly, studies from Asia, the Middle East and other regions have documented bacterial contamination of circulating banknotes where banknotes have been shown to harbor diverse bacterial communities including enteric organisms and opportunistic pathogens (1, 2, 55). The consistency of these findings across geographical regions suggests that currency-associated microbial contamination is a global phenomenon, although the magnitude of contamination may be amplified in settings characterized by intensive cash circulation and limited sanitation infrastructure. The COVID-19 era further renewed global attention to frequently touched public surfaces including banknotes, although most available evidence still indicates contamination potential rather than confirmed transmission events. This is because the detection of bacterial contaminants on currency surfaces does not necessarily equate to a measurable risk of infection transmission. The likelihood of transmission is influenced by multiple factors including microbial viability, inoculum size, survival duration on currency surfaces, frequency of handling, hygiene practices and host susceptibility.

The high contamination burden observed across African studies likely reflects the interaction of cash-dependent informal economies, overcrowded markets, street vending, food-money co-handling and prolonged circulation of worn notes. Earlier African evidence (12) and more recent studies (51, 56, 57) indicate that this problem has persisted across time and circulation settings especially in food-related and high-contact occupational environments. Mechanistically, physically deteriorated paper notes may provide rougher, more absorbent surfaces that favor microbial attachment and persistence whereas polymer notes may carry lower microbial loads because of reduced surface porosity and easier cleaning potential as highlighted by some of the studies (30). Although the presence of bacteria on currency surfaces does not establish direct disease transmission, experimental evidence by Enweani et al. (30) further suggests that currency-associated isolates may possess pathogenic potential as *Escherichia coli*, *Proteus mirabilis* and *Aspergillus* spp. recovered from naira notes induced haematological and histopathological alterations in exposed Wistar rats. These findings suggest that circulating banknotes may serve as important intermediary surfaces linking human, environmental and occupational sources of microbial contamination.

The organism profile also provides insight into likely contamination pathways. The predominance of *Staphylococcus aureus* is consistent with repeated tactile exchange and human skin or nasal carriage while the recovery of *Escherichia coli*, *Salmonella* spp., and *Shigella* spp. points toward fecal contamination and food-handling lapses (7, 11, 58). Opportunistic organisms such as *Klebsiella pneumoniae* and *Pseudomonas aeruginosa* further suggest that some currency-associated bacteria may possess environmental survival traits which may include biofilm formation, stress tolerance and persistence on dry surfaces. Although antimicrobial resistance was not a primary outcome of this review, it is worthy of note that several studies reported resistant bacterial isolates recovered from circulating currency notes suggesting that currency may contribute to the environmental dissemination of resistant organisms through repeated handling and community circulation (11, 19, 40, 49, 50). This observation adds to the One Health dimension, reinforces concerns regarding the community-level spread of antimicrobial resistance and highlights the need for greater integration of environmental reservoirs into antimicrobial resistance surveillance frameworks.

The quantitative synthesis further strengthens the epidemiological significance of currency-associated bacterial contamination across African settings. The pooled meta-analysis demonstrated that contamination affected a substantial proportion of circulating banknotes, suggesting that exposure to contaminated currency surfaces is likely widespread within everyday commercial and community interactions.

Although substantial heterogeneity was observed across the pooled analyses, this variability is likely reflective of genuine epidemiological differences across African circulation settings. Variations in sanitation infrastructure, occupational exposure patterns, climatic conditions, note circulation duration, currency material and microbiological methodologies may all contribute to differences in contamination prevalence across studies. However, the leave-one-out sensitivity analyses consistently demonstrated stability of the pooled estimates following sequential omission of individual studies, indicating that the overall findings were not disproportionately influenced by any single investigation. Similarly, the absence of statistically significant publication bias across the quantitative syntheses further strengthens confidence in the robustness and epidemiological consistency of the meta-analytic findings despite the observed between-study variability.

The findings have practical implications for food safety, infection prevention and public health education. Interventions promoting hand hygiene after handling currency, discouraging simultaneous handling of food and money, replacement of heavily worn banknotes and wider adoption of lower-contamination currency materials such as polymer notes may contribute to reducing microbial transfer through cash circulation networks.

### 4.1 Strengths and limitations

This study has several important strengths that enhance the robustness and relevance of its findings. This represents a comprehensive systematic reviews and meta-analyses evaluating bacterial contamination associated with circulating currency notes across African settings. The review synthesized evidence from multiple African countries and diverse circulation environments including hospitals, markets, transportation systems, food vending sites, and community commercial settings. Another major strength was the integration of both quantitative and narrative evidence thereby providing a broader ecological and public health perspective on currency-associated microbial contamination.

However, several limitations should be acknowledged. Substantial heterogeneity was observed across several pooled analyses. However, such variability is not unexpected in environmental microbiology studies conducted across diverse epidemiological and socioeconomic settings. Inconsistent isolate reporting also limited direct comparability in all the included studies. Additionally, although formal publication bias assessment did not demonstrate statistically significant asymmetry, selective publication of studies with higher contamination burdens cannot be completely excluded. Geographic representation across Africa was uneven, with a large proportion of studies originating from Nigeria and Ethiopia, while several African regions remained underrepresented. The restriction to English-language publications represents an additional limitation. Relevant studies published in French, Arabic, Portuguese, or other languages may have been missed, potentially contributing to the geographical concentration of the evidence base. The findings should therefore be interpreted in light of the uneven geographical representation of the available evidence and the restriction to English-language publications, which may limit their generalizability across Africa.

Despite these limitations, the stability of pooled prevalence estimates during sensitivity analyses supports the overall reliability and epidemiological consistency of the findings.

## Data Availability

The original contributions presented in the study are included in the article/Supplementary material, further inquiries can be directed to the corresponding author.
