# Supplementary material for: Currency as a mobile environmental reservoir of bacterial pathogens in Africa: a systematic review and meta-analysis
Source: Front Public Health. 2026 Oct 19;14:1921421. doi: 10.3389/fpubh.2026.1921421 (PMC13724390; doi:10.3389/fpubh.2026.1921421)
Supplement: Supplementary file 1 [file Data_Sheet_1.PDF]

Supplementary Materials

Contents:
Supplementary Table S1. Database specific search strategies
Supplementary Table S2. Detailed study characteristics
Supplementary Figure S1. Risk-of-bias assessment

Supplementary Table S1: Database specific search strategies

Database	Complete Search Strategy
PubMed	((currency[Title/Abstract] OR "currency note*"[Title/Abstract] OR banknote*[Title/Abstract] OR "paper money"[Title/Abstract] OR "money note*"[Title/Abstract] OR coin*[Title/Abstract] OR cash[Title/Abstract]) AND (bacteria*[Title/Abstract] OR bacterial[Title/Abstract] OR microorganism*[Title/Abstract] OR microbial[Title/Abstract] OR pathogen*[Title/Abstract] OR contamination[Title/Abstract] OR "microbial contamination"[Title/Abstract] OR "bacterial contamination"[Title/Abstract] OR "microbial load"[Title/Abstract] OR "microbiological quality"[Title/Abstract] OR "microbiological assessment"[Title/Abstract]) AND (Africa[Title/Abstract] OR African[Title/Abstract] OR Algeria[Title/Abstract] OR Angola[Title/Abstract] OR Benin[Title/Abstract] OR Botswana[Title/Abstract] OR "Burkina Faso"[Title/Abstract] OR Burundi[Title/Abstract] OR Cameroon[Title/Abstract] OR "Cape Verde"[Title/Abstract] OR "Central African Republic"[Title/Abstract] OR Chad[Title/Abstract] OR Comoros[Title/Abstract] OR Congo[Title/Abstract] OR "Democratic Republic of Congo"[Title/Abstract] OR Djibouti[Title/Abstract] OR Egypt[Title/Abstract] OR "Equatorial Guinea"[Title/Abstract] OR Eritrea[Title/Abstract] OR Eswatini[Title/Abstract] OR Ethiopia[Title/Abstract] OR Gabon[Title/Abstract] OR Gambia[Title/Abstract] OR Ghana[Title/Abstract] OR Guinea[Title/Abstract] OR "Guinea-Bissau"[Title/Abstract] OR "Ivory Coast"[Title/Abstract] OR "Côte d'Ivoire"[Title/Abstract] OR Kenya[Title/Abstract] OR Lesotho[Title/Abstract] OR Liberia[Title/Abstract] OR Libya[Title/Abstract] OR Madagascar[Title/Abstract] OR Malawi[Title/Abstract] OR Mali[Title/Abstract] OR Mauritania[Title/Abstract] OR Mauritius[Title/Abstract] OR Morocco[Title/Abstract] OR Mozambique[Title/Abstract] OR Namibia[Title/Abstract] OR Niger[Title/Abstract] OR Nigeria[Title/Abstract] OR Rwanda[Title/Abstract] OR "Sao Tome and Principe"[Title/Abstract] OR Senegal[Title/Abstract] OR Seychelles[Title/Abstract] OR "Sierra Leone"[Title/Abstract] OR Somalia[Title/Abstract] OR "South Africa"[Title/Abstract] OR "South Sudan"[Title/Abstract] OR Sudan[Title/Abstract] OR Tanzania[Title/Abstract] OR Togo[Title/Abstract] OR Tunisia[Title/Abstract] OR Uganda[Title/Abstract] OR Zambia[Title/Abstract] OR Zimbabwe[Title/Abstract]))
Scopus	TITLE-ABS-KEY ((currency OR "currency note*" OR banknote* OR "paper money" OR "money note*" OR coin* OR cash) AND (bacteria* OR bacterial OR microorganism* OR microbial OR pathogen* OR contamination OR "microbial contamination" OR "bacterial contamination" OR "microbial load" OR "microbiological quality" OR "microbiological assessment") AND (Africa OR African OR Algeria OR Angola OR Benin OR Botswana OR "Burkina Faso" OR Burundi OR Cameroon OR "Cape Verde" OR "Central African Republic" OR Chad OR Comoros OR Congo OR "Democratic Republic of Congo" OR Djibouti OR Egypt OR "Equatorial Guinea" OR Eritrea OR Eswatini OR Ethiopia OR Gabon OR Gambia OR Ghana OR Guinea OR "Guinea-Bissau" OR "Ivory Coast" OR "Côte d'Ivoire" OR Kenya OR Lesotho OR Liberia OR Libya OR Madagascar OR Malawi OR Mali OR Mauritania OR Mauritius OR Morocco OR Mozambique OR Namibia OR Niger OR Nigeria OR Rwanda OR "Sao Tome and Principe" OR Senegal OR Seychelles OR "Sierra Leone" OR Somalia OR "South Africa" OR "South Sudan" OR Sudan OR Tanzania OR Togo OR Tunisia OR Uganda OR Zambia OR Zimbabwe))
Web of Science	TS=((currency OR "currency note*" OR banknote* OR "paper money" OR "money note*" OR coin* OR cash) AND (bacteria* OR bacterial OR microorganism* OR microbial OR pathogen* OR contamination OR "microbial contamination" OR "bacterial contamination" OR "microbial load" OR "microbiological quality" OR "microbiological assessment") AND (Africa OR African OR Algeria OR Angola OR Benin OR Botswana OR "Burkina Faso" OR Burundi OR Cameroon OR "Cape Verde" OR "Central African Republic" OR Chad OR Comoros OR Congo OR "Democratic Republic of Congo" OR Djibouti OR Egypt OR "Equatorial Guinea" OR Eritrea OR Eswatini OR Ethiopia OR Gabon OR Gambia OR Ghana OR Guinea OR "Guinea-Bissau" OR "Ivory Coast" OR "Côte d'Ivoire" OR Kenya OR Lesotho OR Liberia OR Libya OR Madagascar OR Malawi OR Mali OR Mauritania OR Mauritius OR Morocco OR Mozambique OR Namibia OR Niger OR Nigeria OR Rwanda OR "Sao Tome and Principe" OR Senegal OR Seychelles OR "Sierra Leone" OR Somalia OR "South Africa" OR "South Sudan" OR Sudan OR Tanzania OR Togo OR Tunisia OR Uganda OR Zambia OR Zimbabwe))
AJOL	(currency OR "currency notes" OR banknotes OR "paper money" OR coins) AND (bacteria OR bacterial OR microorganisms OR microbial OR pathogens OR contamination OR "microbial contamination" OR "bacterial contamination") AND (Africa OR African)
Google Scholar	("currency notes" OR banknotes OR "paper money" OR coins) AND (bacteria OR bacterial OR microbial OR microorganisms OR pathogens OR contamination OR "microbial contamination") AND (Africa OR African)

Supplementary Table S2: Detailed study characteristics

S/N	Author (Year)	Country	Study Setting	Currency Type	Sample Size	Organisms Identified	Laboratory Identification Method
1	Abdelhakam Ali et al. (2020)[22]	Sudan	Public circulation points in Khartoum	Banknotes and coins	100 samples	*S. aureus*, *K. pneumoniae*, *Enterobacter* spp., *E. faecalis*, *P. aeruginosa*, *E. coli*, *Bacillus* spp.	Culture, Gram stain and biochemical tests
2	Adegoke & Okoh (2011)[32]	Nigeria	Hospital sections in Uyo	Paper naira notes	32 notes	*S. aureus*, *Enterococcus* spp., *E. coli*, *P. aeruginosa*, *S. epidermidis*	Culture, Gram stain and biochemical tests
3	Adegoke et al. (2019)[43]	Nigeria	Community circulation sources in Akure	Naira notes	176 notes	*S. aureus*, *E. coli*, *Pseudomonas* spp., *Klebsiella* spp., *Salmonella* spp., parasites	Culture, biochemical tests and microscopy
4	Adem Hiko et al. (2016)[23]	Ethiopia	Food handlers and milk sellers	Ethiopian Birr notes	434 notes	*Escherichia coli*	EMB culture and biochemical confirmation
5	Agholor et al. (2020)	Nigeria	Polytechnic campus circulation	Naira notes	88 notes	*E. coli*, *B. subtilis*, *S. aureus*, *P. aeruginosa*	Culture, Gram stain and biochemical tests
6	Adonu & Okpara (2025)[51]	Nigeria	Markets, hospitals and transport operators	Naira notes	305 notes	*S. aureus*, *E. coli*, *P. aeruginosa*, *Bacillus* spp., *Salmonella* spp.	Selective culture and biochemical tests
7	Ajayi & Oladele (2019)[52]	Nigeria	Street food vendors	Naira notes	40 notes	*Staphylococcus*, *Klebsiella*, *E. coli*, *Salmonella*, *Shigella* spp.	Selective culture, Gram stain and biochemical tests
8	Akpotu & Funmileso (2021)[53]	Nigeria	Public handlers in Ogun State	Naira notes	240 notes	*E. coli*, *S. typhi*, *S. aureus*, *Bacillus* spp.	Enrichment culture and biochemical tests
9	Amankwah-Kuffour et al. (2015)[8]	Ghana	Street food vending sites	Ghana cedi notes	20 notes	Coliforms, *E. coli*, *Streptococcus* spp., *Salmonella* spp.	APHA microbiological methods
10	Amegovu (2016)[54]	South Sudan	University food restaurants	South Sudanese pound notes	75 notes	Coliforms, *E. coli*, *S. aureus*	ISO swab sampling and selective culture
11	Aminu & Yahaya (2018)[55]	Nigeria	Hospitals and university environment	Naira notes	500 notes	*S. aureus*, *E. coli*, *Proteus*, *Klebsiella*, *Salmonella* spp.	Culture, Gram stain, biochemical tests and AST
12	Anning et al. (2019)[24]	Ghana	Food vendors and transport operators	Ghanaian coins	400 coins	*E. coli*, *Enterobacter*, *Shigella*, *Klebsiella* spp.	Swab enrichment, culture and biochemical tests
13	Asikong et al. (2007)[33]	Nigeria	Markets in Cross River and Imo States	Naira notes	112 notes	*S. aureus*, *Streptococcus* spp., *E. coli*,	Selective culture, Gram stain and AST
14	Attah et al. (2023)[34]	Nigeria	Markets and banks	Naira notes	384 notes	*S. aureus*, *E. coli*, *Pseudomonas* spp.,	Microscopy and bacteriological culture
15	Awe et al. (2010)[35]	Nigeria	Traders and transport workers	Naira notes	NR	*E. coli*, *S. aureus*, *Proteus*, *Klebsiella* spp.	Culture and AST
16	Azza et al. (2019)[56]	Sudan	Food sellers and hospitals	Sudanese banknotes	135 notes	*P. stutzeri*, *E. coli*, *K. pneumoniae*, *A. baumannii*	Culture, PCR and 16S sequencing
17	Bekele Oljira & Kenasa (2018)[44]	Ethiopia	Food handlers and transport workers	Ethiopian birr notes	180 notes	*E. coli*, *S. aureus*, *Salmonella*, *Shigella* spp.	Selective culture and biochemical tests
18	Ezikanyi et al. (2016)[45]	Nigeria	Public circulation points	₦100 notes	72 notes	Fungal spores and parasites	Microscopy and acetolysis
19	Enweani et al (2021)[36]	Nigeria	Banks	Naira Notes	624 notes	*B. subtilis*, *A. faecalis*, *Providencia stuartii, Bacillus amyloliquefaciens,E. coli, P. mirabilis*	Culture and AST
20	Girma et al. (2014)[25]	Ethiopia	Food vendors	Paper currency	100 notes	*Staphylococcus* spp., *Salmonella* spp.	Culture and biochemical identification
21	Igumbor et al. (2007)[26]	South Africa	General circulation	South African rand	240 notes	*S. aureus*, *E. coli*, *Salmonella*, *Shigella* spp.	Culture and biochemical tests

S/N	Author (Year)	Country	Study Setting	Currency Type	Sample Size	Organisms Identified	Laboratory Identification Method
22	Iteku et al. (2020)[57]	DR Congo	Market vendors in Kinshasa	Congolese franc notes	36 notes	*Bacillus* spp., *S. aureus*, *Enterobacter* spp., *E. coli*	Enrichment culture, Gram stain and biochemical tests
23	Kawo et al. (2009)[37]	Nigeria	Markets and transport operators	Naira notes	140 notes	*Bacillus*, *Clostridium*, *Staphylococcus* spp.,	Culture, Gram stain and biochemical tests
24	Kuria et al. (2009)[28]	Kenya	Public handlers in Nairobi	Kenyan coins	40 coins	*E. coli*, *Klebsiella*, *Salmonella*,	Enrichment culture and microscopy
25	Mailafia et al. (2013)[38]	Nigeria	Vendors and transport workers	Naira notes	160 notes	*Bacillus*, *Streptococcus*, *Staphylococcus*, *E. coli*	Culture, Gram stain and AST
26	Mbajiuka et al. (2014)[58]	Nigeria	Fish sellers in Umuahia	Naira notes	63 notes	*Staphylococcus*, *E. coli*, *Pseudomonas*,	Swab culture and microscopy
27	Mbata et al. (2016)[59]	Nigeria	Public handlers in Port Harcourt	Naira notes	56 notes	*S. aureus*, *E. coli*, *Proteus*, fungi, parasites	Culture, microscopy and biochemical tests
28	Mohamed et al. (2020)[46]	Sudan	Public transport and hospitals	Sudanese paper notes	120 notes	*Pseudomonas aeruginosa*	Cetrimide culture and 16S PCR
29	Mofolorunsho et al. (2023)[60]	Nigeria	Students, transport workers, eateries and banks	Nigerian naira notes	64 notes	*E. coli*, *Salmonella* spp., *Shigella* spp.	Selective culture, Gram stain and biochemical tests
30	Morka (2021)[61]	Nigeria	University campus traders	Naira notes	50 notes	*S. aureus*, *E. coli*, *Klebsiella*, *Proteus* spp.	Culture, Gram stain and AST
31	Moses et al. (2018)[41]	Nigeria	Meat sellers	Nigerian paper notes	98 notes	*Escherichia coli*	EMB culture and biochemical tests
32	Neel (2012)[29]	Tanzania	Meat sellers in markets	Tanzanian notes	128 notes	MRSA	Culture, Gram stain and AST
33	Obajuluwa et al. (2023)[62]	Nigeria	Markets, hospitals and transporters	Naira notes	160 notes	*S. aureus*, *E. coli*, *Salmonella*, *Pseudomonas* spp.	Selective culture, biochemical tests and AST
34	Odo et al. (2021)[63]	Nigeria	Fish sellers, food vendors and meat sellers	Nigerian naira notes	72 notes	*S. aureus*, *Salmonella* spp., *E. coli*, *Shigella* spp., *P. aeruginosa*, *Proteus* spp.	Selective culture, Gram stain and biochemical tests
35	Ofoedu et al. (2021)[47]	Nigeria	Food vendors	Naira notes	80 notes	*E. coli*, *Klebsiella*, *Staphylococcus* spp.	Ringer's wash method and biochemical tests
36	Okunye et al. (2023)[42]	Nigeria	Mechanics, butchers, traders and fuel attendants	Nigerian naira notes	160 notes	*S. aureus*, *E. coli*, *P. aeruginosa*, *Bacillus* spp.	Selective culture, Gram stain and biochemical tests
37	Olaleye & Raheem (2024)[64]	Nigeria	Rural and urban communities	Naira notes	112 notes	*Klebsiella*, *Staphylococcus*, *Pseudomonas* spp.,	Conventional culture and fungal morphology
38	Olusola-Makinde et al. (2024)[48]	Nigeria	Market circulation sources	Naira notes	60 notes	*S. aureus*, *E. coli*, *S. typhi*, *P. aeruginosa*	Culture, biochemical tests and 16S sequencing
39	Otokunefor et al. (2023)[65]	Nigeria	University population	Naira notes	50 notes	*S. aureus*, *E. coli*, *Proteus*, *Klebsiella* spp.	Culture, biochemical tests and AST
40	Saadabi et al. (2010)[30]	Sudan	Markets and transport operators	Sudanese banknotes	NR	*E. coli*, *Klebsiella*, *Shigella*, *S. aureus*, fungi	Culture, biochemical tests and microscopy
41	Saleh et al. (2018)[31]	Egypt	Hospitals, markets and food handlers	Egyptian notes and coins	120 samples	CoNS, *S. aureus*, *E. coli*, *A. baumannii*, fungi	Culture, Gram stain, biochemical tests and AST
42	Sivalingam & Dola (2021)[66]	Ethiopia	Markets, taxi drivers, fruit sellers and hotels	Ethiopian paper notes	240 notes	*S. aureus*, *E. coli*, *Shigella* spp., *Salmonella* spp.	Selective culture, Gram stain, biochemical tests and AST
43	Wodajo & Kabeta (2022)[13]	Ethiopia	Butcher houses and restaurants	Ethiopian Birr notes	108 notes	*S. aureus*, *S. epidermidis*, *S. hyicus*	MSA culture, biochemical tests and AST

S/N	Author (Year)	Country	Study Setting	Currency Type	Sample Size	Organisms Identified	Laboratory Identification Method
44	Yar (2020)[10]	Ghana	Shops and transport handlers	Ghanaian paper notes	70 notes	*E. coli*, *S. aureus*, *Klebsiella*, *Salmonella*, *Proteus*, *Shigella* spp.	Swab culture, biochemical tests and AST
45	Iyevhobu et al. (2023)[50]	Nigeria	Banks, markets, restaurants and fuel stations	Nigerian paper naira notes	40 notes	*E. coli*, *S. aureus*, *P. mirabilis*, *Streptococcus* spp., *Klebsiella* spp.	Culture, Gram stain and biochemical tests
46	Imarenezor et al. (2018)[49]	Nigeria	Food vendors, traders, cobblers and meat vendors	Nigerian naira notes	40 notes	*S. aureus*, *E. coli*, *P. aeruginosa*, *Bacillus* spp., *Salmonella* spp., *Klebsiella* spp., *Streptococcus* spp.	Culture, Gram stain and biochemical tests

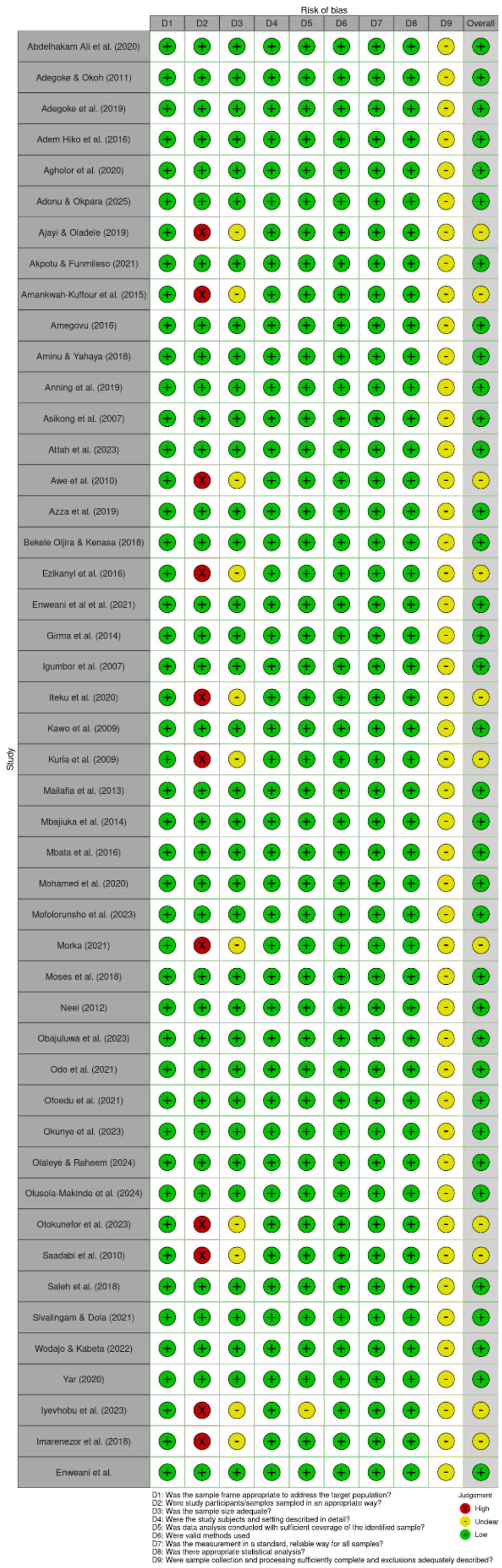

Supplementary Figure S1: Risk of Bias Assessment

ccccccccccc
